# Per-Oral Immunization with Antigen-Conjugated Nanoparticles followed by Sub-Cutaneous Boosting Immunization Induces Long-Lasting Mucosal and Systemic Antibody Responses in Mice

**DOI:** 10.1371/journal.pone.0118067

**Published:** 2015-02-24

**Authors:** Savannah E. Howe, Vjollca H. Konjufca

**Affiliations:** Department of Microbiology, Southern Illinois University, Carbondale, Illinois, United States of America; New York State Dept. Health, UNITED STATES

## Abstract

Food or water-borne enteric pathogens invade their hosts via intestinal mucosal surfaces, thus developing effective oral vaccines would greatly reduce the burden of infectious diseases. The nature of the antigen, as well as the mode of its internalization in the intestinal mucosa affects the ensuing immune response. We show that model protein antigen ovalbumin (Ova) given per-orally (p.o.) induces oral tolerance (OT), characterized by systemic IgG1—dominated antibody response, which cannot be boosted by sub-cutaneous (s.c.) immunization with Ova in complete Freund’s adjuvant (CFA). Intestinal IgA generated in response to Ova feeding diminished over time and was abrogated by s.c. immunization with Ova+CFA. Humoral response to Ova was altered by administering Ova conjugated to 20 nm nanoparticles (NP-Ova). P.o. administration of NP-Ova induced systemic IgG1/IgG2c, and primed the intestinal mucosa for secretion of IgA. These responses were boosted by secondary s.c. immunization with Ova+CFA or p.o. immunization with NP-Ova. However, only in s.c.-boosted mice serum and mucosal antibody titers remained elevated for 6 months after priming. In contrast, s.c. priming with NP-Ova induced IgG1-dominated serum antibodies, but did not prime the intestinal mucosa for secretion of IgA, even after secondary p.o. immunization with NP-Ova. These results indicate that Ova conjugated to NPs reaches the internal milieu in an immunogenic form and that mucosal immunization with NP-Ova is necessary for induction of a polarized Th1/Th2 immune response, as well as intestinal IgA response. In addition, mucosal priming with NP-Ova, followed by s.c. boosting induces superior systemic and mucosal memory responses. These findings are important for the development of efficacious mucosal vaccines.

## Introduction

The majority of viral, bacterial, and parasitic infections occur at mucosal surfaces, thus developing effective mucosal vaccines would greatly decrease the burden of infectious diseases. This task has however been challenging, mainly due to the poor stability, uptake, and immunogenicity of mucosally-administered antigens. As a result, very few mucosal vaccines are currently licensed for use in humans [1]. Oral vaccines are especially convenient for mass-immunizations, since they are preferred over parenteral injections and eliminate the use of needles and syringes [2]. To be effective, oral vaccines must be efficiently internalized at mucosal surfaces and induce antigen-specific effector, as well as memory B and T cell responses. Especially important for protection against pathogens and their toxins are mucosal antibodies, which can neutralize mucosal antigens and limit their access to the internal milieu [3]. Secretory IgA, a predominant antibody in intestinal secretions, can bind to and neutralize microorganisms and toxins, preventing them from making contact with and crossing the epithelial cell barrier [4,5]. Specifically, intestinal IgA was shown to neutralize cholera toxin [6,7], reduce motility of *Salmonella* [8], as well as decrease the ability of *Shigella* to invade the intestinal epithelium [9]. In addition, oral transfer of specific IgA antibodies was shown to protect mice against bacterial infections such as *S*. *typhimurium* [10,11], *V*. *cholera* [12], *S*. *flexneri* [13], and *H*. *felis* [14]. In addition to aiding in the “trapping” of antigens in the intestinal mucus, IgA is also important for expelling antigens from the internal milieu into the intestinal lumen via transcytosis, as well as transporting lumen antigens into underlying lymphoid tissues for the initiation of immune responses [15,16,17,18]. Although parenteral vaccination induces systemic antibodies and protection against some mucosal pathogens such as HPV, polio and influenza viruses [19,20], mucosal vaccination induces systemic, and most importantly, local mucosal antibodies that can offer protection against mucosal pathogens such as HIV, rotavirus, norovirus, *V*. *cholera*, and *Mycobacterium* spp. [21,22,23,24,25]. Therefore, the efficacy of an oral vaccine will in great part depend on the vaccine’s ability to induce long-lasting production of antibodies at mucosal surfaces. In addition, to increase the efficacy of vaccine formulations, various prime-boost immunization strategies have been used [26]. Prime-boost immunization regimen influences localization and the strength of the immune response induced, thus vaccine efficacy [27]. The immunogenicity of many vaccine formulations depend on their co-administration with adjuvants. However, there are safety concerns associated with the use of most effective adjuvants. Similarly, live attenuated vaccine strains that have been developed for mucosal immunization raise concerns that attenuated strains might revert to virulence, trigger, exacerbate autoimmune diseases, or cause disease in immunocompromised individuals [28].

To overcome some of these challenges, nano-scale particles (such as liposomes, ISCOMs, virus-like particles, etc.) have become increasingly popular as vehicles for the delivery of antigens and drugs [29]. NPs of various sizes have been engineered of biodegradable materials and can be impregnated with or conjugated to multiple antigens, and thus potentially be safe while inducing immunity to multiple pathogens. NPs larger than 200 nm have been mainly used for antigen delivery due to their ability to carry larger amount of antigen cargo [30,31,32]. However, smaller NPs can penetrate the mucus barrier and are internalized at mucosal surfaces more efficiently than larger NPs [33,34,35]. We showed that intestinal epithelial cells efficiently internalize p.o. administered 20 and 40 nm NPs, which are then transported to the draining mesenteric lymph nodes (MLNs) [36]. Here we demonstrate that NP-conjugated antigen administered p.o. reaches the internal milieu in an immunogenic form and induces systemic and mucosal antibodies. In addition, we show that mucosal priming with NP-Ova is necessary for a mixed systemic Th1/Th2 immune response. Moreover, mucosal priming with NP-Ova, followed by s.c. boosting immunization was necessary for induction of long-lasting serum IgG1 and IgG2c, as well as intestinal IgA. These findings have implications for the development of mucosal vaccines and prime-boost immunization strategies. In addition, this work will aid in the understanding of fundamental mechanisms that govern immune responses to orally acquired antigens.

## Materials and Methods

### Ethics statement

This study was carried out in strict accordance with the recommendations in the Guide for the Care and Use of Laboratory Animals of the National Institutes of Health. The protocol was approved by the Southern Illinois University Institutional Animal Care and Use Committee (Protocol Number: 13–057). Animals were housed in centralized AAALAC-accredited research animal facilities, staffed with trained husbandry, technical, and veterinary personnel.

### Animals, reagents, and antibodies

For these studies six to eight week-old male and female C57BL/6 mice (Jackson Laboratories) were used. Chicken Ova (Sigma) was used as a model protein antigen. Carboxylate-modified fluorescent polystyrene nanoparticles (20 nm, Invitrogen) were conjugated to Ova and every batch of conjugated NPs was analyzed by dot-blot as described previously [36]. Biotinylated rabbit anti-Ova antibodies (Thermo Scientific) in combination with streptavidin-FITC (eBioscience) were used to detect Ova and NP-Ova on dot blots. Goat anti-mouse IgG1, IgG2c, and IgA antibodies conjugated to alkaline phosphatase (AP) (Southern Biotechnology) were used for determination of antibody titers in sera and fecal extracts of immunized mice as described previously [37].

### Administration of Ova and NP-Ova to the mice

For p.o. immunizations mice were fasted for 4 h, then administered 200 μl PBS (control), Ova (25 mg/200 μl PBS), NP-Ova (0.25 mg/200 μl PBS), or an equivalent dose of Ova (0.25 mg/200 μl PBS) via a gastric gavage using a round-tip needle on days 0, 3, 6, and 8. In total, mice received 0 mg Ova (control), 100 mg, and 1mg Ova (either as NP-Ova or soluble Ova). For immunizations NPs were diluted 1:10 in PBS, from an original concentration of 2% (wt/wol). At day 28 after p.o. inoculation, mice were s.c. injected with 300 μg Ova in CFA (Sigma). In other experiments mice were primed either p.o. with NP-Ova as described above or s.c. with 200 μl of NP-Ova, then boosted p.o. with 200 μl of NP-Ova diluted in PBS at 10% from an original NP concentration of 2%.

### Collection of fecal pellets and blood samples

Before immunizations and every week thereafter, fecal pellets were collected from each mouse and diluted in PBS containing 0.02% sodium azide (Sigma) to a final concentration of 100 mg dry matter/ml of PBS. Diluted fecal pellets were homogenized and then centrifuged at 10,000 × g for 10 minutes. Supernatant devoid of fecal debris was collected and stored at -20°C until further analysis. Blood samples were collected via the tail vein using a 30 g needle, and serum was stored at -20°C until further analysis.

### Determination of Ova-specific antibody titers in sera and fecal extracts using ELISA assay

Flat-bottomed 96-well plates were coated with 100 μl of 50 μg/ml Ova (Sigma) solution in coating buffer (0.02 M Na_2_CO_3_/0.07 M NaHCO_3_ in H_2_O, pH 9.6) and allowed to incubate overnight at 4°C. After the unbound antigen was removed, wells were then blocked for 1 h at 37°C with 200 μl of blocking buffer (0.2% porcine gelatin (Sigma) in PBS). Although bovine serum albumin (BSA) is often used for ELISA assays, to avoid experimental errors stemming from cross-reactions between BSA and Ova-specific antibodies, as well as between Ova and anti-BSA antibodies [38], porcine gelatin was used. After blocking, plates were washed three times with PBS containing 0.05% Tween-20 (Sigma) and 0.02% sodium azide (Sigma) using an automated plate washer (BioTek, ELx50). After washing, 200 μl of sample (serum or fecal extract, diluted in blocking buffer) were added to the first column of wells and then diluted into successive wells of blocking buffer and allowed to incubate overnight at 4°C. After overnight incubations, plates were washed three times, and then to each well 100 μl of AP-conjugated goat anti-mouse IgG1, IgG2c, or IgA, diluted 1:2000 in blocking buffer were added and allowed to incubate for 2 h at room temperature. Plates were then washed three times, and AP activity was assayed by adding 100 μl of 1 mg/ml AP substrate (Sigma) then incubating for 20 minutes at room temperature, protected from light. The reaction was stopped with 25 μl of 3 M NaOH and the absorbance was read at 405 nm using a plate reader (BioTek, Epoch). Antibody titers are expressed as log_10_ value of the highest reciprocal dilution that yielded an OD value twice that of a negative control.

### Statistical analysis

Each experiment was repeated twice. Data were analyzed using ANOVA procedures of SAS software. Group means were separated using Student’s t-test or Tukey’s multiple comparison procedure and were considered significantly different at P<0.05. Data are expressed as the mean ± standard deviation of the mean.

## Results

### P.O. administration of NP-Ova induces serum IgG1/IgG2c and intestinal IgA, while Ova feeding induces serum IgG1-dominated antibodies and short-lived intestinal IgA

**Fig 1:**
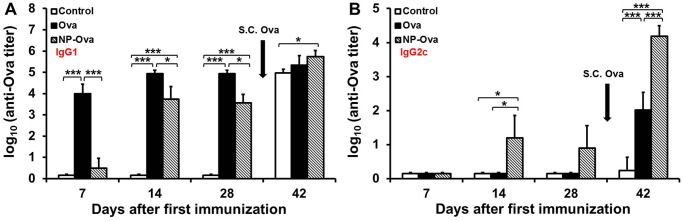
Antibody responses in sera of mice primed p.o. with soluble Ova, NP-Ova or PBS. Mice were administered PBS (control), soluble Ova, or 20 nm NP-Ova p.o. at day 0, 3, 6 and 8, then s.c. boosted with 300 μg Ova+CFA at day 28 (arrow). Ova-specific serum IgG1 (A) and IgG2c (B) antibody titers are expressed as log_10_ titer values, with the titer being the highest dilution showing an absorbance value twice that of the background. Data collected from 10 mice per group (2 separate experiments) are expressed as the mean ± SD of the mean. Group means were separated using Tukey’s multiple comparison procedure and were declared significantly different at p<0.05 (*) and p<0.01 (***).

**Fig 2:**
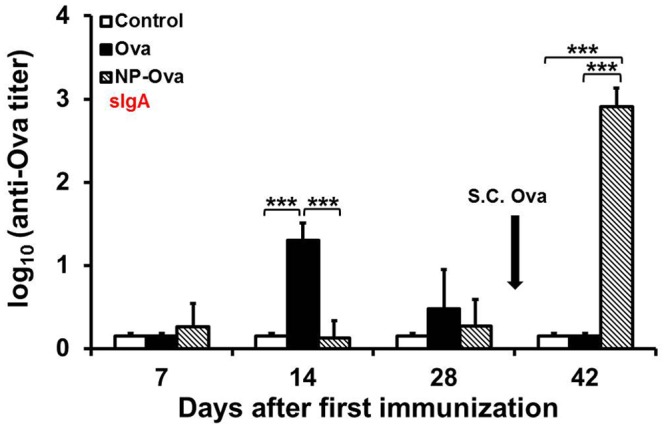
Intestinal IgA responses in mice primed p.o. with soluble Ova, NP-Ova or PBS. Mice were administered PBS (control), soluble Ova, or 20 nm NP-Ova p.o. at day 0, 3, 6 and 8, then s.c. boosted with 300 μg Ova+CFA at day 28 (arrow). Fecal extracts were assayed for Ova-specific IgA using ELISA assay. Ova-specific IgA titers are expressed as log_10_ titer values, with the titer being the highest dilution showing an absorbance value twice that of the background. Data collected from 10 mice per group (2 separate experiments) are expressed as the mean ± SD of the mean. Group means were separated using Tukey’s multiple comparison procedure and were declared significantly different at p<0.05 (*) and p<0.01 (***).

We examined whether administering Ova conjugated to NPs p.o. would induce antigen-specific immunity, rather than OT. OT generated to dietary antigens is characterized by reductions in T-cell functions, suppression of serum IgE, Th1-dependent IgG2a (IgG2c in C57BL/6 mice), as well as mucosal IgA responses [39,40,41]. Thus as a control the immunization protocol with a high dose of Ova shown to induce OT [42] was used in order to examine whether Ova delivery via NPs abrogates OT. In addition, a group of mice were fed a low dose of soluble Ova (similar to the dose given via NPs) in order to exclude the possibility that immune responses observed in NP-Ova immunized mice are due to the antigen dose. Another feature of OT is the suppression of a systemic, as well as intestinal immune response to the subsequent antigen exposure. Therefore, at day 28 after the last p.o. administration of soluble Ova, NP-Ova or PBS (control), mice were injected s.c. with Ova+CFA. Mice that were fed Ova exhibited significantly higher serum IgG1 titers at day 7, 14, and 28 compared to control mice and mice that were given NP-Ova p.o. (Fig. 1A). As expected, no Ova-specific serum IgG1 was detected in control mice at day 7, 14, or 28 (Fig. 1A). S.c. injection of Ova+CFA at day 28 induced serum IgG1 in control mice and significantly boosted the serum IgG1 titers in mice given NP-Ova (p<0.05), but not the mice fed soluble Ova (p<0.11) (Fig. 1A). In contrast to this, mice that were given NP-Ova exhibited significantly elevated serum IgG2c titers at day 14 compared to control and Ova-fed mice (Fig. 1B). S.c. immunization significantly boosted the serum IgG2c titers of NP-Ova (p<0.01) and Ova-primed (p<0.01) mice, while control mice exhibited no serum IgG2c titers before or after s.c. immunization (Fig. 1B). Mice primed with NP-Ova had significantly higher serum IgG2c titers at day 42 compared to the titers of Ova-fed and control mice (p<0.01) (Fig. 1B). To examine the changes of Ova-specific intestinal IgA over time, fecal pellets were collected from individual mice and assayed for the presence of IgA. No appreciable amount of IgA was detected in fecal extracts of control mice and NP-Ova-primed mice at day 7, 14, or 28 (Fig. 2). In contrast, mice fed soluble Ova exhibited significantly elevated fecal IgA titers at day 14 after p.o. administration (p<0.01) (Fig. 2). However, these titers did not persist and were not different from titers of control or NP-Ova-primed mice by day 28 (Fig. 2). S.c. immunization with Ova+CFA at day 28 significantly boosted the IgA titers only in fecal extracts of mice immunized p.o. with NP-Ova, but abrogated intestinal IgA in Ova-fed mice. Control mice primed p.o. with PBS and s.c. immunized with Ova+CFA at day 28 exhibited no IgA in fecal extracts at any time point examined (Fig. 2). Mice that were fed a dose of soluble Ova that was comparable to the amount of Ova given via NP-Ova exhibited serum IgG1 and IgG2c that were slightly lower than the titers of mice fed a high dose Ova (not shown). In mice fed a low dose Ova, IgA was detectable in fecal extracts at day 14 after immunization and was comparable to the IgA titers in extracts of mice fed high dose Ova (25 mg/dose). In addition, s.c. injection with Ova+CFA did not boost the intestinal IgA of mice fed a low dose Ova, like seen in mice fed a high dose Ova. Mice that were p.o. primed with NP-Ova had significantly higher IgA (p<0.01) compared to mice fed a low (not shown) and a high dose of Ova after s.c. immunization (Fig. 2).

### P.o. priming with NP-Ova is necessary for inducing isotype switch, leading to a polarized IgG1/IgG2c systemic immune response, and intestinal IgA

**Fig 3:**
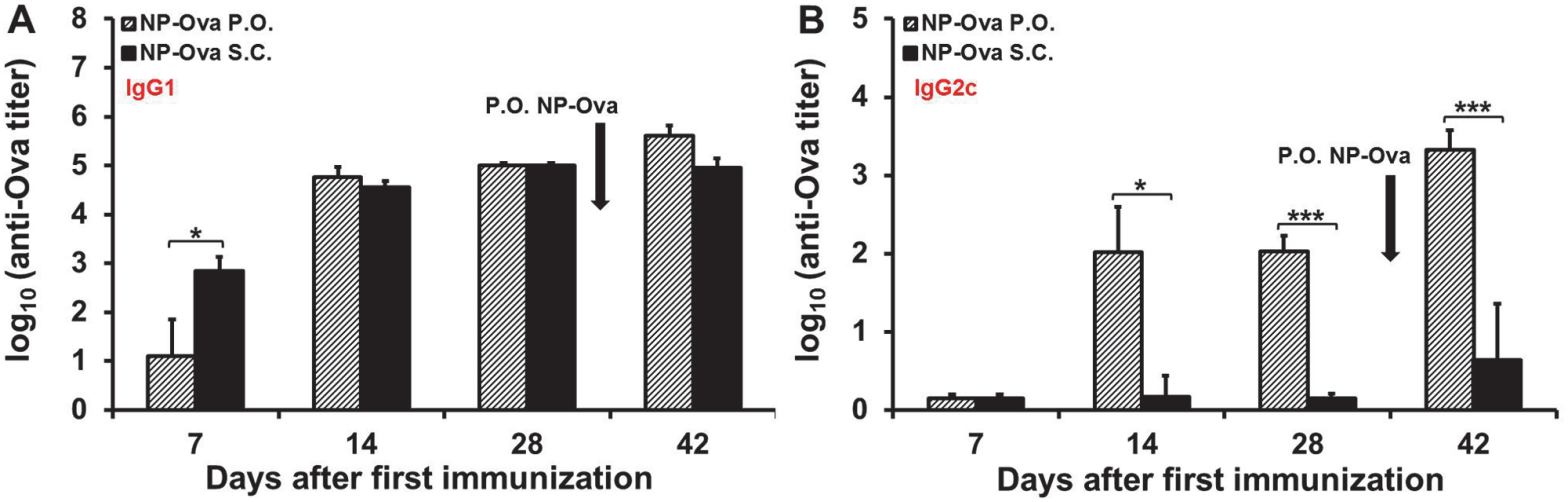
Serum antibody titers of mice boosted p.o. with NP-Ova after either p.o. or s.c. priming with NP-Ova. Groups of 5 mice were p.o. or s.c. primed with NP-Ova, then p.o. boosted with NP-Ova at day 28 (arrow). Ova-specific serum IgG1 (A) and IgG2c (B) titers are expressed as log_10_ titer values, with the titer being the highest dilution showing an absorbance value twice that of the background. Data are expressed as the mean ± SD of the mean. Group means were separated using Student’s t-test and were declared significantly different at p<0.05 (*) and p<0.01 (***).

**Fig 4:**
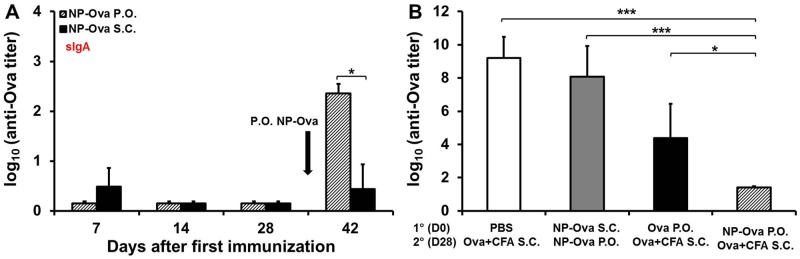
Induction of intestinal IgA and serum IgG2c antibody response depends on the immunization route that was used for priming. (A) Groups of 5 mice were p.o. or s.c. primed with NP-Ova, then p.o. boosted with NP-Ova at day 28 (arrow). Intestinal IgA titers are expressed as log_10_ titer values, with the titer being the highest dilution showing an absorbance value twice that of the background. Group means were separated using Student’s t-test and declared significantly different at P<0.05 (*). (B) Ratio of OVA-specific IgG1:IgG2c in sera of mice at 42 days after the first immunization. Mice were primed p.o. (with PBS, Ova, NP-Ova) or s.c. (with NP-Ova), then boosted with Ova+CFA s.c. or NP-Ova p.o. Data are expressed as the mean ± SD of the mean. Group means were separated using Tukey’s multiple comparison procedure and declared significantly different at P<0.05 (*) or P<0.01 (***).

We then tested whether p.o. priming with NP-Ova was absolutely needed for a systemic Th1/Th2 polarization (IgG1/IgG2c) and for induction of intestinal IgA. For this, two groups of mice were first immunized either p.o. or s.c. with NP-Ova, then p.o. boosted with NP-Ova. At day 7 s.c.-primed mice had significantly higher serum IgG1 titers compared to the p.o.- primed mice, however both groups had comparable serum IgG1 titers at days 14, and 28 after immunization, which were slightly increased by a second p.o. immunization only in p.o.-primed mice (p<0.26) (Fig. 3A). Mice primed p.o. with NP-Ova exhibited substantial serum IgG2c titers by day 14, which were significantly higher (p<0.01) compared to the titers of s.c.-primed mice at days 14, 28 and 42 (Fig. 3B). As in the first set of experiments, a significant amount of IgA was observed in fecal extracts of p.o.-primed mice only after a second p.o. NP-Ova administration at day 28 (Fig. 4A). At day 42 the p.o. primed and p.o. boosted mice had significantly higher intestinal IgA compared to the mice primed s.c. and boosted p.o. with NP-Ova (p<0.05). Unexpectedly, at day 7 and 42, mice that were primed s.c. with NP-Ova had measurable IgA in pooled samples of fecal extracts. Analysis of individual serum and fecal extract samples collected at day 7 and 42 revealed that only 1 of five mice of the s.c.-primed and p.o.-boosted group, had some serum IgG2c and intestinal IgA, skewing the overall averages of the group (Figs. 3B and 4A). Examination of serum IgG1:IgG2c ratios at day 42 revealed that s.c. priming with Ova+CFA (control group) or NP-Ova led to IgG1-dominated response and IgG1:IgG2c ratios that were significantly higher compared to the IgG1:IgG2c ratios of mice p.o. primed with NP-Ova (Fig. 4B). In addition, mice primed p.o. with soluble Ova and boosted s.c. with Ova+CFA had significantly higher IgG1:IgG2c ratios compared to the mice primed p.o. with NP-Ova (Fig. 4B). Mice primed p.o. with NP-Ova then boosted either s.c. with Ova+CFA, or p.o. with NP-Ova (not shown) had lowest IgG1:IgG2c ratios, indicating a strong Th1 polarization. At day 42 the IgG1:IgG2c ratios between these two groups did not differ significantly (not shown).

### Systemic and mucosal antibody titers induced by p.o. NP-Ova administration, followed by s.c. boost with Ova+CFA remain high for extended periods of time

**Fig 5:**
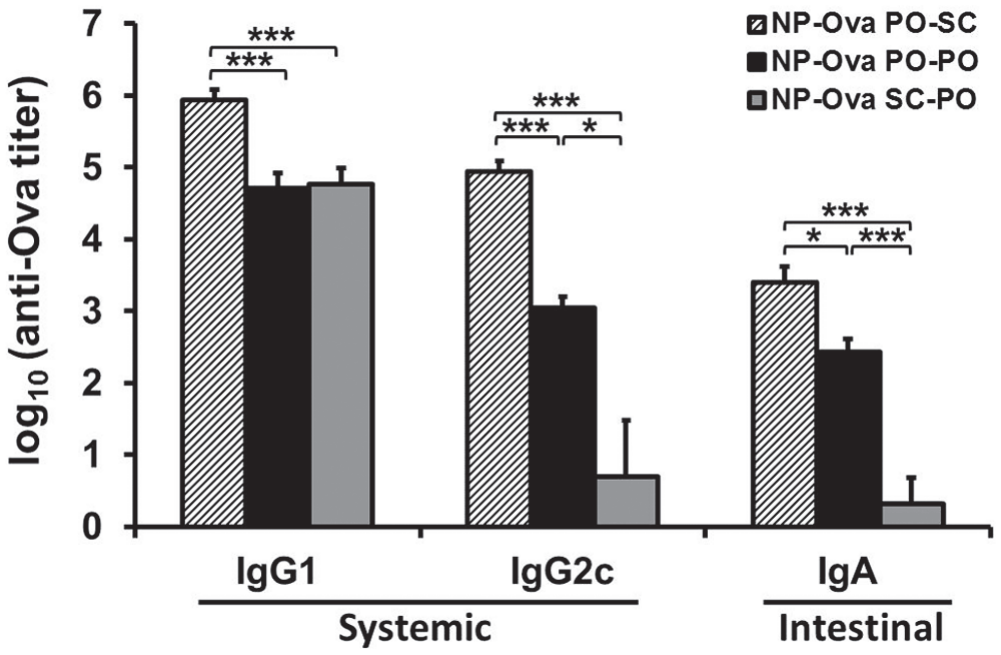
Mouse serum IgG1, IgG2c, and fecal IgA antibody titers at 6 months (180 days) after priming immunization. Groups of 5 mice were: p.o. primed with NP-Ova, then s.c. boosted with Ova+CFA at day 28 (PO-SC); p.o. primed with NP-Ova, then p.o. boosted with NP-Ova (PO-PO); or s.c. primed with NP-Ova, followed by p.o. boost with NP-Ova (SC-PO). Ova-specific antibody titers are expressed as log_10_ titer values, with the titer being the highest dilution showing an absorbance value twice that of the background. Data are expressed as the mean ± SD of the mean. Group means were separated using Tukey’s multiple comparison procedure and declared significantly different at P<0.05 (*) or P<0.01 (***).

Analysis of serum and fecal samples collected at 6 months after priming revealed that the titers of Ova-specific serum IgG1and IgG2c, as well as intestinal IgA were significantly higher in mice p.o. primed with NP-Ova and s.c. boosted with Ova+CFA compared to mice that were p.o. primed and p.o. boosted with NP-Ova (Fig. 5). In addition, mice p.o. primed and boosted with NP-Ova had significantly higher IgG2c and intestinal IgA compared to s.c. primed, p.o. boosted mice (Fig. 5). Interestingly, at 6 months after priming, mice that were p.o. primed with NP-Ova and s.c. boosted with Ova+CFA had significantly elevated serum IgG2c and intestinal IgA titers compared to day 42 titers (Table 1). In contrast, in mice primed and boosted p.o. with NP-Ova serum IgG1 titers decreased significantly by 6 months after priming (p<0.01). There was also a decrease, albeit non-significant, in serum IgG2c titers, while intestinal IgA titers did not change appreciably (Table 1). In mice s.c. primed and p.o. boosted with NP-Ova, serum IgG1, IgG2c, and intestinal IgA titers did not differ significantly between day 42 and 180 (Table 1).

**Table 1:** Systemic and mucosal Ova-specific antibody titers at day42 and 180 after priming immunization.

Immunization Strategy	Isotype	Log_10_ Ova-specific antibody titers^a^		
		Day 42	Day 180	*p-v*alue
1° NP-Ova P.O. 2° Ova+CFA S.C.	IgG1	5.74 ± 0.6	5.94 ± 0.3	0.3
	IgG2c	4.19 ± 0.6	4.94 ± 0.1	0.002
	sIgA	2.91 ± 0.5	3.41 ± 0.1	0.04
1° NP-Ova P.O. 2° NP-Ova P.O.	IgG1	5.61 ± 0.4	4.71 ± 0.3	0.01
	IgG2c	3.32 ± 0.5	3.05 ± 0.1	0.3
	sIgA	2.35 ± 0.4	2.43 ± 0.4	0.8
1° NP-Ova S.C. 2° NP-Ova P.O.	IgG1	4.95 ± 0.4	4.77 ± 0.4	0.5
	IgG2c	0.64 ± 1.4	0.70 ± 1.5	0.9
	sIgA	0.44 ± 0.9	0.32 ± 0.7	0.8

^a^Data are expressed as the mean ± SD of the mean. Group means were separated using Student’s t-test.

## Discussion

The nature of the antigen (thus the vaccine formulation) affects its uptake in the intestinal mucosa, transport to the deeper lymphoid tissues, and consequently its immunogenicity. Larger antigens (bacteria, particles, etc.) are mainly internalized by M cells that are found in the epithelium overlying Peyer’s patches (PP) and isolated lymphoid follicles. Bacteria can also be internalized by CD11c^+^ [43], CX3CR1^+^ [44], and CD103^+^ [45] dendritic cells (DCs) of the lamina propria, which can extend their dendrites between epithelial cells of the small intestine. In contrast, soluble protein antigens that reach the small intestine undigested can enter the lamina propria via goblet cell-associated passageways [36,46], and are transferred to CD103^+^ DCs. Transport of protein antigens to the MLNs by CD103^+^ DCs of the lamina propria was shown to be necessary for induction of OT [42]. Howe et al. showed that 20 and 40 nm NPs were internalized not only by the epithelial cells overlying PP, but also by epithelial cells overlying the villi [36]. In addition, within the lamina propria of the villi, NPs were frequently observed co-localizing with the CD11c^+^ DCs [36] and tolerogenic CD103^+^ DCs (unpublished data). We investigated whether p.o. administration of Ova model antigen conjugated to 20 nm NPs would induce Ova-specific immune responses, rather than OT that is seen with administration of soluble Ova. In addition, we examined how the routes of priming and secondary boosting immunizations affect ensuing systemic and mucosal antibody responses. We show that p.o. administration of soluble Ova induces systemic IgG1-dominated response which is not boosted by a subsequent s.c. Ova administration. In addition, Ova feeding induced short-lived Ova-specific intestinal IgA. This response was reminiscent of the intestinal IgA response to an auxotrophic *E.coli* strain, which was rapidly abrogated following an exposure to commensal microbes [47]. Thus, much like the repertoire of intestinal IgA that appears to reflect the major microbial species present in the gut at a given time [47], the intestinal IgA repertoire might also reflect dominant dietary intestinal antigens. Mucosal IgA response to soluble Ova (low and high dose) also lacks immune memory and classical prime-boost characteristics, as s.c. injection of Ova+CFA did not boost Ova-specific intestinal IgA. In contrast, Ova conjugated to 20 nm NPs induced a mixed serum IgG1/IgG2c antibody response and primed the intestinal mucosa for secretion of IgA. Unlike antibody titers induced by soluble Ova, serum IgG1/IgG2c, as well as intestinal IgA induced by p.o. NP-Ova were substantially boosted by subsequent s.c. Ova+CFA or p.o. NP-Ova administration. In agreement with findings of others [48] we show that NP-Ova administered s.c. are just as efficient as CFA in inducing Ova-specific antibodies. In addition, NPs do not produce local or peripheral inflammatory reactions that are often observed with CFA or other adjuvants. Another very important observation is that mucosal priming with NP-Ova induced a strong Th1/Th2 polarization, while s.c. priming induced mainly a Th2 type immune response, as shown by analysis of serum IgG1 and IgG2c antibody titers. The predominance of serum IgG1 and the lack of serum IgG2c and intestinal IgA in mice s.c. primed with NP-Ova indicates that Ova delivery via NPs per se does not induce isotype switching. The antigen administration route that was used for priming immunization influences the Th1 or Th2 skewing, and strongest Th1 polarization was seen in mice p.o. primed with NP-Ova. In addition, intestinal IgA was observed only in mice that were primed via mucosal surfaces (p.o.) with either Ova or NP-Ova. IgA isotype switching can occur in PP, MLNs, and possibly in the lamina propria of the small intestine [49]. All these anatomic locations can harbor microbial PAMPs originating from commensal microflora [50], thus antigen presentation in this context may be responsible for isotype switching, resulting in systemic IgG1, IgG2c, and intestinal IgA. Others have shown that MyD88 signaling is necessary for induction of IgG2c to thymus-independent antigens (such as Ova), and that the development of a primary Th1 response and IgG2c requires MyD88 activation of both DCs and B lymphocytes [2]. The lack of MyD88 was also shown to cause an impairment of IgA production in Peyer’s patches, indicating that signaling via toll-like receptors plays a key role in IgA-mediated mucosal immunity [51,52]. In two separate experiments, s.c. immunization with Ova+CFA alone did not induce an appreciable amount of serum IgG2c two weeks after immunization. Similarly, mice s.c. primed with NP-Ova alone had IgG1-dominated humoral response, although in 1 of the 5 mice some IgG2c and intestinal IgA was observed. Small NPs (20 nm-200 nm) and 30 nm virus-like particles administered intra-dermally were shown to freely drain into the local LNs [48,53], while large NPs mostly remained within the injection site [53]. Therefore, it is conceivable that a small fraction of NPs s.c. injected on the back of a mouse may have also reached the MLNs, where isotype switching might have occurred. Induction of IgA responses in the MLNs after s.c. immunization was previously demonstrated, leading to a suggestion that there is a functional link between the skin and mucosal tissues [54].

Generation of mucosal immunological memory, the most important consequence of vaccination, was shown to be possible using cholera toxin as an antigen [55]. However, rapidly waning mucosal immunity, a main limitation of many mucosal vaccines including the oral poliovirus vaccine [56], indicates that mucosal memory may be short-lived. Mucosal IgA induced after p.o. immunization of mice with antigen-loaded 3 μm particles without adjuvants, was decreased by about 40% and 60% by week 7 and 8 after immunization respectively [30]. In a similar study, salivary IgA antibody titers were reduced by almost 90% by week 13 after immunization [31], indicating that secretion of intestinal IgA induced by p.o. immunization with large particles is short-lived. We show that a single p.o. immunization with NPs alone is not sufficient to induce high titers of IgA in fecal extracts. However, s.c. immunization of the p.o. primed mice boosted the intestinal IgA titers, which further increased and were highest at 6 months after p.o. priming. In addition, a p.o.-p.o. immunization with NP-Ova also boosted the intestinal IgA, which unlike systemic IgG1 and IgG2c did not appreciably decline by 6 months after priming. This finding is in contrast to the findings of others [30,31] and the discrepancy in our results might be the difference in the size of NPs used for immunization, as it is well documented that the uptake of NPs in the intestines is inversely correlated with their size. In addition, the size of NPs was also shown to play a role in the magnitude and the quality of the immune response, as smaller NPs were shown to be more efficient than larger NPs in inducing humoral and cell-mediated immunity, as well as in protecting mice from tumors [48].

Induction of a mixed Th1/Th2 immune response and mucosal immune memory are essential for the efficacy of a mucosal vaccine. Effective Th1 responses are important for protection against mucosal pathogens such as *L. monocytogenes*, *Salmonella* spp., *M. tuberculosis*, *H. pylori*, etc. [57,58,59]. Th2 responses support antibody production such as mucosal IgA and systemic IgG, which are essential for protection against *Salmonella* spp, *Shigella* spp., *Vibrio cholera* and its toxin, rotavirus, norovirus, influenza virus, poliovirus etc. [1,6,7,8,9,10].

Better understanding of how the NP-conjugated antigen is transported to the deeper lymphoid tissues, processed, and presented to T lymphocytes will be important for designing NP-based mucosal vaccines. In addition, it will aid our understanding of how the increasingly prevalent dietary NPs might play a role in priming the intestinal mucosa to innocuous dietary antigens. The use of NPs for vaccine development has several advantages over other approaches. NPs are internalized efficiently by intestinal epithelial cells and do not require adjuvants to be effective, thus raising no safety concerns. Biodegradable NPs, made of non-toxic materials can be loaded into digestible capsules in order to protect the antigen cargo from degradation. In addition, capsules can be impregnated with NPs conjugated to multiple antigens, thus increasing the immunogenicity of vaccine formulations. More work is needed in order to explore these possibilities. Although a needle-free p.o. administration of NP-based mucosal vaccines is especially appealing for mass immunizations, a secondary s.c. boosting immunization appears to be essential for induction of long-lasting systemic and mucosal antibodies.
